# The Significance of Toll-Like Receptors in the Neuroimmunologic Background of Alcohol Dependence

**DOI:** 10.3389/fpsyt.2021.797123

**Published:** 2022-01-12

**Authors:** Agnieszka Czerwińska-Błaszczyk, Edyta Pawlak, Tomasz Pawłowski

**Affiliations:** ^1^Division of Psychotherapy and Psychosomatic Medicine, Wroclaw Medical University, Wroclaw, Poland; ^2^Laboratory of Immunopatology, Department of Experimental Therapy, Hirszfeld Institute of Immunology and Experimental Therapy, Polish Academy of Sciences, Wroclaw, Poland

**Keywords:** toll-like receptors, TLR, alcohol dependence, sterile inflammation, immune activation

## Abstract

Toll-like receptors (TLR) are a group of protein belonging to the family of Pattern Recognition Receptors (PRR) which have the ability to distinguish between an organism's own antigens and foreign ones and to induce immunological response. TLR play a significant part in non-specific immunity but at the same time they are also a vital element linking non-specific response to the specific one. A growing number of data seems to indicate that the non-specific immunity mechanisms affect the development and sustenance of alcohol addiction. Alcohol damages the organism's cells not only directly but also through an increase inintestinal permeability which induces innate immune response of peripheral blood cells. The signaling pathway of Toll-like receptors located on the surface of brain immune cells intensifies the inflammatory reaction and, through modifying gene expression of proinflammatory factors, unnaturally supports it. This overly protracted “sterile inflammatory reaction” positively correlates with alcohol craving affecting also the functioning of the reward system structures and increasing the risk of relapse of alcoholism. Recurrent alcoholic binges sensitize the microglia and cause an escalation in inflammatory reaction which also leads to neurodegeneration. The induction of innate immunity signaling pathways exposes clinical symptoms of alcohol addiction such as increased impulsivity, loss of behavioral control, depressive-anxiety symptoms and cognitive dysfunctions. Traditional methods of treating alcohol addiction have tended to focus predominantly on reducing symptoms which—given the frequency of relapses—seems insufficient. The aim of the present paper is to discuss the role of toll-like receptors as elements of the immunity system which, together with the nervous system, plays a crucial part in the pathogenesis of alcohol addiction. We also wish to present pharmacotherapeutic perspectives targeted at the neuroimmunological mechanisms of alcohol addiction.

## Introduction

Alcohol addiction, a chronic disease, is characterized by a constant need to seek, acquire, and consume a particular substance, i.e., alcohol. This need is beyond a person's control and can lead to physical ailments or negative emotional states when access to the substance is blocked (1).

As a global public health problem, excessive alcohol consumption causes social, economic and health damage. According to the World Health Organization, as many as 2.348 billion people aged 15 and over drink alcohol (which is 43% of the world's population). Men consume about twice as much alcohol as women (53.6 vs. 32.3%), which is the cause of more than 200 different diseases (2). Due to alcohol-related diseases and injuries, life expectancy is estimated to decrease by 0.9 years by 2050. In Eastern and Central Europe, the rate is even higher at 1.6 years (3).

The etiopathogenesis of alcohol dependence is multifactorial and the clinical picture is complex and varied. In *1987*, Robert Cloninger attempted a comprehensive comparative analysis of these *two* types of alcoholism, in which he linked the addict's personality traits with the activity of neurotransmitter systems and, as a result, distinguished the following types of alcoholism: type I—occurring mainly in women around the age of 25, without genetic background, and type II—occurring in men and genetically conditioned (4). Type I alcoholics are introverts prone to anxiety and depression, often raised in strict homes and usually conforming to social norms. Type II alcoholics are extroverts with antisocial tendencies (5). Cloninger linked these personality types to differences in neurotransmitter functioning, noting that Type I alcoholics have a severe deficiency in dopaminergic transmission and an increased serotonergic transmission. Quite frequently they are neurotic and passive-dependent personalities who rely on alcohol for reducing symptoms of social anxiety and not for thrill seeking. Since type I alcoholics experience a stronger fear of what the consequences of their drinking addiction may be, they suffer from less acute social and legal implications of their alcoholism. Type II alcoholics—while suffering from a deficit in serotonergic transmission—have no problems with dopaminergic transmission, and sensation seeking is a unique feature of their condition (4, 5). They tend to exhibit impulsive and aggresive *behaviors* which result from a low level of fear of the consequences of their drinking. That is also why they are prone to experience greater and faster alcohol-induced losses (6).

Cloninger's theory laid the foundation for the psychobiological model of the development of alcohol dependence that is still in place today. In the integrative approach, the development of alcohol dependence is determined by biological, psychological, and environmental factors. Only the interaction of all three factors leads to the development of the disease; the independent presence of single factors is insufficient for the development of alcohol dependence. In this understanding, alcohol dependence is not a genetic disease, although an individual's biological conditions have a significant impact on his or her susceptibility to developing an addiction. Specific biological conditions associated with high tolerance to alcohol consumption and high intensity of its euphoric effect are associated, among others, with changes in genes encoding alcohol metabolizing enzymes (aldehyde dehydrogenase, ALDH) and in genes responsible for neurotransmission in the central nervous system. Dysfunction of ALDH leads to increased sensitivity to alcohol, which consequently prevents alcohol abuse. Thus, the presence and degree of ALDH dysfunction may determine different patterns of alcohol consumption in different individuals.

The level of ALDH is related to gender, in women the level of ALDH is 70–80% lower than in men, which consequently increases the effect of alcohol on the female body because they feel the effects of alcohol faster and after smaller doses. An additional factor related to alcohol metabolism is the volume of body fat and circulating blood, as well as the effect of estrogen. What's more, the action of ALDH weakens with age so that older people are more susceptible to feel the negative effects of alcohol (7). Personality traits such as impulsivity, timidity, depression, compulsion, low tolerance for monotony and boredom, and increased susceptibility to frustration are also markers for the development of addiction (8, 9).

From an immunological point of view—alcohol, which belongs to the group of xenobiotics with psychoactive properties, stimulates an innate (non-specific) immune response whose mechanisms play an important role in the pathogenesis of the transition from the substance abuse phase to the dependence phase by stimulating the reward system in the brain (2). Furthermore, overactivity of the immune system generates and sustains the negative consequences of alcohol and other psychoactive substance use, most notably neurodegeneration, mediated by the mechanism of neuroimmunity, which “sensitizes” immune cells in the brain, such as microglia and astrocytes, through the consumption of psychoactive substances. This leads to their negative overactivity called “reactive microglia”/“reactive astrogliosis” (8), which can cause overproduction of neurotoxic inflammatory factors and ultimately lead to apoptosis of self-replicating cells and neurodegeneration.

Studies of “sterile inflammation” in the brain have led to the identification of innate immune receptors (based on their response to specific pathogens) as pattern recognition receptors (PRRs), recognizing and responding not only to specific molecular patterns present on foreign (exogenous) pathogens—so-called pathogen-associated molecular patterns (PAMPS), but also to endogenous signaling molecules associated with cell damage, degeneration, or stress—so-called danger-associated molecular patterns (DAMPs) (10). Five classes of PRRs are currently characterized: (1) TLRs, (2) C-type lectin receptors, (3) nucleotide-binding domain oligomerization receptors (NOD-like receptors), (4) RIG-I-like receptors, and (5) AIM2-like receptors (11). Of these receptors, the most thoroughly characterized and well-studied group is the TLR family of PRRs, which activate signaling cascades to promote expression of proinflammatory cytokines.

Increased TLR-node immune signaling in the brain has been documented to lead to epigenetic modifications, changes in synaptic plasticity as well as loss of neuronal cell populations, resulting in cognitive and emotional dysfunction. Furthermore, Toll-like receptor signaling in cortico-limbic circuits is modified by alcohol and stress in a manner with promotion of progression through stages of addiction.

Therefore, the aim of this paper is to describe in detail and summarize the existing knowledge regarding the importance of toll-like receptors as elements of the immune system, which, together with the central nervous system, play a key role in the etiopathogenesis of alcohol dependence.

## Toll-Like Receptors

Of the 13 TLR receptors identified to date, the human genome contains 11 active ones, either localized on the cell surface or intracellularly. TLRs on the cell surface include TLR1, TLR2, TLR4, TLR5, TLR6, and TLR10, whereas intracellular TLRs are located in the endosome and include TLR3, TLR7, TLR8, TLR9, TLR11, TLR12, and TLR13. Surface-localized TLRs mainly recognize components of microbial cell membranes such as lipids, lipoproteins, and proteins—TLR4 is stimulated by ethanol, according to the study. TLR1, TLR2 and TLR6 recognize a wide range of PAMPs; TLR5 recognizes bacterial flagellin. Human TLR10 cooperates with TLR2 to recognize listeria-derived ligands. It can also detect influenza A infection (12).

After binding to the ligand, the key activator of the signaling cascade is Toll-IL-1-receptor (TIR), the cytoplasmic terminal domain of the TLR molecule, so named because of its affinity for the interleukin-1 type 1 receptor (IL-1R1). TIR binds to specific adaptor proteins, the most important of which is MyD88 (myeloid differentiation primary response gene 88), as its presence determines the signaling pathway. For most toll-like receptors, MyD88 is the sole adaptor, except for TLR3, whose adaptor is the TRIF (TIR domain-containing adaptor inducing IFN-beta) protein, and TLR4, which induces both a MyD88-dependent and independent pathway through the use of TRIF and TRAM (TRIF-related adaptor molecule) proteins (13).

The cascade of activation of individual transcription factors as well as the increase in gene expression for proinflammatory cytokines is determined by the TLR signal transduction pathway; in other words, the choice of signaling pathway determines its end products, influencing the course of the inflammatory response, which is an adaptive mechanism to the situation in which the organism becomes infected with pathogens. Depending on the type of pathogen, a specific inflammatory response pattern is activated.

In the immune system response to bacterial infection, an important role is played by the MyD88-dependent signaling pathway, which is initiated by binding of the adaptor protein MyD88 to the TIR domain of the toll-like receptor. This complex then activates interleukon-1 receptor-associated kinase (IRAK), which through tumor necrosis factor receptor-associated factor (TRAF) initiates the mitogen-activated protein kinase (MAPK) signaling cascade, leading to activation of nuclear factor kappa-light-chain-enhancer of activated B-cells (NF-κB). The transcription factor NF-κB influences the expression of genes responsible for the secretion of proinflammatory cytokines: interleukin-1B (IL-1B), tumor necrosis factor alpha (TNF-alpha), interleukin-6 (IL-6), interleukin-8 (IL-8) (14). By stimulating the expression of major histocompatibility complex (MHC) protein, NF-κB activation also promotes the process of antigen presentation by T lymphocytes. In the central nervous system environment, 50-fold higher concentrations of this factor have been documented in glial cells compared to its levels in neurons, which may indicate a predisposition of glial cells to generate a more intense inflammatory response. This is confirmed by the expression level of TNF-alpha as an end product of NF-κB activation, which is approximately 300 times higher in brain immune cells than in neurons (15). Given that serum TNF-alpha levels positively correlate with the duration of alcohol consumption, and that this factor crosses the blood-brain barrier, the induction of the inflammatory response following ethanol intoxication may occur at both peripheral and central levels in this particular pathomechanism (16).

In turn, the immune system response to viruses is based on a TRIF-dependent signaling cascade in which TLRs attach to the TRIF/TCAM-1 adaptor protein, which in turn triggers the signaling cascade of TANK-binding kinase 1 (IKKε) and TBK1 kinase, ultimately leading to the synthesis of type 1 interferons, mainly IFN-β.

Both the MyD88-dependent and independent toll-like receptor signaling pathway can be initiated in response to the activity of their endogenous antagonists, the DAMP structures, in the case of the ethanol toxicity damage discussed in this paper. The pattern of inflammatory response in the brain triggered by toll-like receptor activation depends on several factors.

First, toll-like receptors are able to regulate the intensity of the inflammatory response by cooperating with each other, as confirmed by *in vitro* studies in mice where it was shown that simultaneous stimulation of several toll-like receptors (TLR4 plus TLR2, TLR4 plus TLR9, and TLR2 plus TLR9) in microglia by individual ligands results in an enhanced inflammatory response compared to when only one toll-like receptor is stimulated (17).

Second, toll-like receptors can extinguish each other's activity, thereby protecting the body from an excessive inflammatory response that could lead to septic shock and death. This was demonstrated in an experiment in which additional stimulation of TLR7 arrested the unfolded inflammatory response triggered by activation of several combinations of TLRs, resulting in reduced expression of pro-inflammatory factors in microglia, such as TNF-α, IL-6, CXCL1 and IL-10, and the neuronal activation product nitric oxide NO (14).

Since the expression of TLRs is differentiated in individual cells of the nervous system and dependent on their localization, it can therefore cause neurodegeneration as a result of the developed inflammatory response, both by activation of microglia (TLR2, TLR4) and by indirect damage to nerve cells, without the presence of microglia (TLR7) (14).

However, the severity of the inflammatory response in the brain depends not only on the combination of individual TLRs, but also on the ligands through which they are activated (14).

## Immunological System in the Brain

Glial cells, including microglia and macroglia consisting of astrocytes, oligodendrocyte lineage cells, and lining cells, are regulators of the brain's immune response (18).

Astrocytes are responsible for maintaining the blood-brain barrier by regulating the migration of lymphocytes and monocytes into the CNS. They also integrate signals from neuronal synapses and regulate the secretion of neurotransmitters into the synaptic area. In addition, they are involved in the inflammatory response in the brain by increasing the expression of genes for pro-inflammatory factors such as IL-1 beta, IL-6, TNF-alpha and INF-y (19). Oligodendrocytes are responsible for myelination of nerve cell axons and since myelin has not only an insulating function but is also metabolically active. Oligodendrocytes are also transporters of metabolic products, thus providing energetic support for neurons (20, 21). Lining cells are found in the spinal cord and ventricles of the brain, which are responsible for the production, excretion and circulation of cerebrospinal fluid (18, 22).

Microglia are a type of primary cells of the innate immune system of the brain and spinal cord that migrate to these sites in the early embryonic stage of the fetus and, while remaining in a resting form, monitor the tissue microenvironment by modulating synaptic transmission directly (19, 23) or through interactions with astrocytes (20, 24). Microglia respond to perturbations of homeostasis in the CNS that can be induced by exogenous factors such as bacterial, viral, and/or fungal pathogens or their toxins, xenobiotics, or endogenous factors that include compounds released by dead or damaged cells. Microglia cells can be found in all areas of the brain, but their highest concentration is found in the black matter, basal ganglia, and hippocampus (16). This brain safety system, composed of microglia and astrocytes, is activated by a signaling cascade triggered by toll-like receptors (TLRs), a key mechanism in the pathogenesis of addiction.

## Consumption Model vs. Type of Immune System Activation

Alcohol consumption model—heavy drinking or chronic alcohol consumption affects immune function by altering TLR activity in microglia and macrophages. Single high doses of ethanol (450 mM) have been shown to inhibit the pro-inflammatory response of the immune system, which is associated with disruption of TLR3 (25), TLR4 (26, 27), TRL2 and TLR9 (28) signaling. Low/moderate doses of alcohol (50 mM) consumed chronically stimulate the TLR4 signaling pathway, resulting in the induction of pro-inflammatory factors that increase MHC protein expression while inhibiting T cell proliferation. Thus, despite activation of the innate immune system, there is no initiation of an adaptive threat response (29). In an attempt to explain the effects of ethanol on toll-like receptor inflammatory responses, the lipid raft hypothesis has been proposed as one potential solution. Lipid rafts are domains of the cell membrane containing many sterols and sphingolipids and their characteristic proteins. They take part in the regulation of cell membrane fluidity, signal transduction and apoptosis (30). Ethanol triggers the migration of TLRs into the lipid rafts of CNS immune cells to initiate inflammatory signaling, but in a dose-dependent manner, alcohol can modulate cell membrane structure and function, resulting in disruption of signaling pathways. It has been confirmed that consumption of high doses of alcohol (100 mM) increases the fluidity of the cell membrane by modifying its content, which disrupts the recruitment of TLR4 and TLR2 to lipid rafts (31, 32). Alcohol in low to moderate doses (10–50 mM) in turn promotes the recruitment of TLRs to the cell membrane (33). Long-term alcohol consumption increases cholesterol levels in cell membranes, leading to an increased number of ethanol uptake sites, resulting in impaired ethanol action and explaining the increased tolerance with chronic drinking. Many microorganisms also utilize lipid rafts for entry into the host system and for intracellular survival and replication. By impairing the recruitment of TLRs to lipid rafts, ethanos disrupts the pro-inflammatory signaling pathway, further altering lipid raft structure to prevent cell activation by LPS. This may explain why chronic alcohol users are more prone to inflammatory processes and infections. This may also be due to weakening of the intestinal barrier and stimulation of the immune system by LPS, but macrophages in chronically alcohol-exposed mice have been shown to be more sensitive to inflammatory stimuli than cells not exposed to alcohol (34). Chronically administered ethanol abolishes the immunosuppressive effects of single doses of alcohol and “sensitizes” immune cells (35).

## The Role of Toll-Like Receptors in Alcohol Addiction

### Dysfunction of the Intestinal Barrier (Leaky Gut) and Activation of Hepatitis

The triggering of the inflammatory response after alcohol consumption begins at the level of the intestines, where the so-called GALT—gut-associated lymphoid tissue—functions. GALT is connected to cell membranes and together they build the intestinal immune system. It houses 75% of the lymphoid cells of the entire immune system, and up to 80% of immunoglobulins are produced in the gut. When the cell membranes of the intestinal system come into contact with antigens entering the body from the outside with nutrients, the body's immune system is activated and immune memory develops (36). The surface of the cell membranes in the intestines is populated by beneficial bacterial flora, which ensures the proper functioning of the immune system. Each person has their own unique microbiome profile, which is conditioned and shaped already in fetal life and then changes during life under the influence of various factors, including alcohol. The gut microbiota is part of the gut-brain axis, which communicates with the CNS through various pathways, including the immune system, *via* tryptophan metabolism, the vagus nerve, and the enteric nervous system. This particular signaling pathway plays an important role in generating negative effects of alcohol consumption, such as systemic inflammatory response, immune deficiency, hepatitis, and mood disorders.

The current state of research confirms that high-dose alcohol consumption (at least 2–3 g/kg of gastric doses EtOH) leads to the breakdown of intestinal tissue and the release of bacterial endotoxins such as lipopolysaccharide (LPS) and peptidoglycan (PGN) from the intestinal lumen into the general circulation. TLR activation on peripheral blood mononuclear cells (PBMCs) leads to activation of the immune system, resulting in systemic inflammation. This in turn explains the presence of a chronic “sterile” inflammatory response in alcohol-dependent individuals, manifested by elevated inflammatory factors such as TNFα, interleukins (IL)-1β, IL-6, IL-8, IL-10, without bacterial or viral infection (37).

Chronic alcohol consumption also leads to quantitative and qualitative changes in the intestinal microflora, or dysbiosis. Leaky intestinal barrier has been shown to be a very early sign of dysbiosis and occurs within two weeks of alcohol consumption, while actual intestinal dysbiosis occurs later, about 8–10 weeks after ethanol intake (38). The gut microflora builds a communication axis between the gut and the brain through hormonal, neural, and immune signaling, thereby influencing neurotransmitters in the CNS. Probiotic bacteria such as Lactobacillus and Bifidobacterium—*via* the vagus nerve—have been confirmed to affect GABAB receptors, which play a key role in mood disorders and anxiety symptoms in alcoholics. Dysbiosis interferes with the development of lymphoid tissue in the gut, which in turn interferes with immunoglobulin production. Patients with alcohol dependence suffer from abnormal antigen production, which is manifested by elevated levels of immunoglobulins (IgG, IgA and IgM) with a decrease in the number of B lymphocytes. Additionally, they exhibit elevated levels of immunoglobulins (IgG, IgA, and IgM). Additionally, they show elevated levels of over-activated T lymphocytes, which positively correlates with alcohol consumption. This indicates that disturbances in the gut microflora have a significant impact on the dysregulation of the immune system and immune impairment so common in alcoholics (39). It appears that both alcohol consumption and intestinal dysbiosis are necessary to cause leaky gut.

As a result of bacterial endotoxin leakage through a perforated gut wall into the portal circulation, signaling cascade of many Toll-like receptors in the liver is initiated (TLR1, 2, 4, 6, 7, 8 and 9), and the activated Kuppfer cells secrete a range of proinflammatory factors, which creates the pathogenetic base for alcohol-induced liver damage. Mice without MyD88, a crucial adapter of the toll-dependent signaling pathway, did not exhibit an increased activity of alanine aminotransferase in blood serum and did not have an increased level of liver triglycerides, which are the early markers of alcohol-induced liver inflammation and steatosis (40). As a result of cell atrophy and liver tissue damage the level of HGB1 (High mobility group box 1, HMGB1) in peripheral blood rises. As an inflammation mediator, the protein activates macrophages, monocytes, neutrophiles and Th lymphocytes, intensifying its chemotaxis and the synthesis of proinflammatory factors (IL-1, IL-6, IL-8). HMGB1 is also the direct antagonist of TLR and—as DAMP—it links with TLR2 and TLR4, which leads to an increase in NF-κB expression and the release of proinflammatory cytokines. Through linking in with TLR4 on neutrophiles, it can also stimulate the production of reactive oxygen forms by NADPH oxidase (38, 41).

Thus, this indicates that not only is the innate immune system involved in the development of addiction, but it also exacerbates negative health implications such as chronic liver damage caused by ethanol.

### Alteration of the Blood–Brain Barrier and Activation of CNS Inflammation

Alcohol-activated cells of the peripheral immune system migrate to the CNS due to loss of blood-brain barrier (BBB) integrity, which in turn activates inflammation in the CNS. Reduction of BBB permeability occurs through activation of the TLR4 signaling pathway, which increases the activity of MMP-9, a kinase belonging to MAPKs that has the ability to reduce basement membrane and extracellular matrix proteins. In addition, glial cells, particularly astrocytes and microglia, also show an increase in MMP-9 due to the induction of pro-inflammatory cytokines such as IL-1β and TNF-α. No changes in BBB permeability under ethanol were observed in TLR4-deficient mice, indicating that activation of this receptor plays an important role in the cooperation of the peripheral and central immune systems in generating a systemic inflammatory response. That the inflammation generated in the periphery is sustained by central mechanisms is also evidenced by the fact that patients with alcohol dependence continue to have elevated levels of IL-6 and TNF-α after a three-week abstinence. This prolonged immune system response—which persists despite abstinence from alcohol—positively correlates with alcohol craving, which seems to underscore the importance of the immune system in generating clinical symptoms of addiction (42–44).

Blocking the signaling cascade of the TRIF-dependent kinase pathway led to decreased alcohol consumption (45). NF-κB as a product of TRIF-dependent TLR3 activation reaches from the cytoplasm to the cell nucleus, leading to the expression of genes responsible for the secretion of proinflammatory cytokines such as IL6, IL10 Il-1 β, TPA, TNF-α and IFN-β (46). Blocking NF-κB reduces ethanol-induced induction of pro-inflammatory genes in the hippocampus of alcohol-dependent subjects (47).

### Alteration of Neurotransmitters in CNS

The effects of ethanol on GABAergic transmission are due to its effects on processes in the central nucleus of the amygdala (CeA), which is a cluster of GABAergic neurons. In alcohol addiction, this area of the brain plays a special role because it is involved in learning the importance of reward in response to a stimulus, in compulsive drinking, and is also responsible for the occurrence of anxiety in alcohol withdrawal, which is a strong stimulus to return to heavy drinking. By stimulating TLR4, alcohol enhances GABAergic transmission, which is regulated by the GABA A receptor (GABAAR). The function of TLR4 in CeA involves both direct binding of TLR4 to the L2 subunit of the GABA receptor and stimulation of inflammatory factor release. TLR4 activated in CeA enhances GABAergic transmission in neurons. The interaction of GABAAR with ethanol occurs mainly through a presynaptic and indirectly postsynaptic (neurotransmitter release) mechanism. Immediately after ethanol ingestion, receptor inhibition occurs at the presynaptic level to restore GABA transmission homeostasis. Increased GABAergic transmission after alcohol consumption results in improved mood and anxiolytic effects. With chronic alcohol consumption, the body develops tolerance to the sedative effects of ethanol, and abrupt cessation of drinking or alcohol withdrawal leads to stimulation of neurotransmitter systems and development of alcohol withdrawal syndrome (AWS). Increased expression of TLR4 in CeA and its interaction with GABAAR results in overactivation of GABA L2 receptors, which inhibits neuronal excitability and leads to increased alcohol consumption (binge drinking). Chronic repeated alcohol consumption and withdrawal leads to changes in brain activity resulting in persistent hyperactivity (48–55).

Activation of the TLR4 pathway, whose end product includes IL-1β, plays an important role in generating the effects of alcohol dependence. TLR4 activation leads to increased expression of IL-1β in CeA. Human genetic studies have shown that polymorphisms in genes encoding receptors for IL-1β may be responsible for a greater susceptibility to develop alcohol dependence. Furthermore, IL-1β, through its effects on GABAergic transmission, exacerbates anxiety-like behaviors during alcohol withdrawal. Studies in alcohol-fed mice have shown that TLR4 deficiency protects the body from excessive glial activation, induction of inflammatory cytokines, and apoptosis (56–59).

IL-1β-receptor antagonism reduces binge drinking and inhibits ethanol-induced central nervous system inflammation (46). Ethanol stimulates the reward system, which includes the prefrontal cortex (PFC), striate body, caudate nucleus, amygdala, and hippocampus. The reward system in the brain is largely associated with dopaminergic transmission and is based in the ventral tegmental area (VTA), which contains the cell bodies of dopaminergic neurons. Under physiological conditions, activation of the reward system is responsible for sensing stimuli and experiencing pleasure and bliss, achieved through stimulation of the VTA and increased dopamine output, which facilitates further pleasure seeking.

When the reward system is stimulated by hunger, thirst, or sex, dopamine release is gradual and steady, unlike addiction-induced stimulation in which the frequency of dopamine discharge is much greater and occurs in stages (60). The specific discharge pattern of VTA neurons in the addiction situation depends on the presentation of the reward. In the initial phase of alcohol consumption, when alcohol is an unexpected reward, i.e., not conditioned by stimuli that predict its delivery, the discharge of dopaminergic neurons has a higher frequency, leading to a transient increase in dopamine. If, on the other hand, the reward is expected, i.e., it was preceded by a conditioned stimulus, the response of dopaminergic neurons occurs immediately after the stimulus is acted upon and is appropriate to the magnitude of the expected reward, meaning that dopamine is released in response to the announcement of the reward rather than as a reaction to its actual delivery. If the reward is not delivered at the expected time, dopamine output drops below baseline levels, motivating the individual to resort to other strategies to obtain the reward (61, 62).

Thus, during the initial phase of alcohol use, there is a process of increased dopamine release to promote reward, whereas with prolonged alcohol exposure, a decrease in dopamine transmission is revealed. There are several hypotheses to explain this phenomenon, including decreased frequency of dopamine release from the VTA (63), increased dopaminergic transmitter activity in the striatum (64), or increased GABAergic transmission, which may inhibit the activity of dopaminergic neurons (65). HMGB1 as a direct TLR antagonist is one of the proinflammatory factors that mediate chronic microglia activation and lead to dopaminergic cell death (66, 67).

### Anxiety Symptoms and Cognitive Disfunction

Anxiety symptoms after alcohol withdrawal occur when the peripheral inflammatory response is suppressed, while activation of the immune system in HF continues, as evidenced by elevated levels of cytokines (Il- 1 β, Il- 17) and chemokines (MIP-1a - macrophage inflammatory protein-1a, MIP-1a and CX3CL1- chemokine - C-X3-C motif, ligand 1, CX3CL1) in the striatum, an area of the brain involved in the development of addiction and serum. That is, the brain area involved in the development of addiction and in blood serum. The importance of the immune system in generating anxiety symptoms resulting from alcohol withdrawal is demonstrated by the fact that elimination of TLR4 and/or TLR2 in mice inhibited the production of inflammatory mediators in the striatum and prevented the onset of anxiety symptoms (68).

TLR activation in ethanol-influenced glial cells induces an inflammatory response and leads to neuronal cell apoptosis, which correlates with the occurrence of cognitive impairment. Additionally, neuronal apoptosis exacerbates inflammation in the CNS through the release of DAMPs and activation of the immune system signaling cascade. Cognitive impairment in alcohol-dependent individuals is due to the loss of nerve cells on the one hand and the presence of demyelinating lesions on the other. Post-mortem studies of individuals who chronically consumed alcohol showed that they had lower brain mass resulting from a reduction in white matter and reduced levels of N-acetylaspartate (NAA, a marker of neuronal viability) compared to healthy individuals. Studies in mice have also shown that alcohol-induced activation of TLR4 causes a reduction in proteins involved in myelination, including proteolipid protein (PLP), myelin basic protein (MBP), myelin glycoligodendrocytes, and myelin-associated glycoprotein. Impaired levels of synaptic and myelin proteins cause ultrastructural changes in the prefrontal cortex, an area of the brain that coordinates higher-order cognitive processes and executive functions. Alcohol-induced myelination disorders, especially those arising during adolescence, a period of intense neurocognitive development, may affect attention and working memory functions and impair decision-making processes, which positively correlates with a tendency to choose immediate rewards as opposed to delayed rewards, also increasing impulsivity. The occurrence of cognitive dysfunction in alcohol-dependent individuals is also due to lower levels of dopamine, which manifests as difficulty in attributing proper meaning to newly learned conditioned stimuli and leads to decreased motivation to experience pleasure while sober, resulting in the occurrence of alcohol craving (69–73).

## Conclusions and Future Perspectives

Alcohol addiction is a chronic and progressive disease of a neurobiological background which affects the human emotion regulation system thus influencing a person's perception of the world and reactions to various *situ*ations. Personality traits related to genetic and environmental factors as *well* as specific changes in the functioning of neurotransmitters determine an individual's model of alcohol consumption. Alcohol causes extensive damage not only through a direct toxic impact on organs, but also through inducing neuroimmunisation which is a maladaptive mechanism occurring in conditions of chronic cell stress or a prolonged inflammatory reaction.

Inducing the toll-like *signaling* cascade manifests itself through an inflammation in the Central Nervous System and through immunosuppression in the periphery which explains the greater susceptibility of alcoholics to infections. A sterile inflammatory reaction in the brain caused by alcohol, through its impact on the reward system, is involved in generating axial symptoms of addiction such as substance craving and loss of *behavioral* control.

Inflammatory *signaling* in the brain is not extinguished immediately once a person starts abstaining from the substance, which promotes *behaviors* related to reward seeking and increases the risk of a relapse.

The *signaling* cascade of toll-like receptors plays a vital role also in generating negative affective states and cognitive dysfunctions observed in people addicted to the substance.

Relapses are the basic problem in treating alcohol addiction, and the hitherto applied treatment methods such as psychotherapy, substitution treatment or pharmacotherapy with opioid receptor antagonists are all largely based on symptom elimination and do not constitute a full-on causal treatment which is why they frequently prove insufficient.

Thus, contemporary trends in addiction pharmacology should focus on treating the causes of alcoholism and not just its clinical symptoms. Treatment should account for how alcohol affects the patient's genetic, molecular, cellular, anatomic and *behavioral* mechanisms.

Promising outcomes seem to be achieved with medicines inhibiting microglia activity which have a neuroprotective effect, such as *e.g*., ibudilast, a *non*-specific phosphodiesterase inhibitor. Its neuroprotective effect on neurons and glial cells is dual, *i.e*., it consists both in inhibiting the production of inflammatory mediators—both cytokines and reactive oxygen forms, and in increasing the production of neurotrophic factors in the activated microglia (74–76).

Further research into toll-like receptors appears to be paving the way for new trends which should focus more on the neuroimmunologic foundations of addiction, thus constituting a precious alternative to conventional treatment, both in terms of therapeutic effectiveness and improvement of patients' quality of life.

## Author Contributions

AC-B manuscript concept and plan, literature analysis, and preparation of the manuscript. TP manuscript plan, literature analysis, preparation of the manuscript, and substantive supervision. EP manuscript plan, literature analysis, and preparation of the manuscript. All authors had equal participation in the creation of the manuscript and have approved the final article.

## Funding

This work was funded by a Wroclaw Medical University grant SUB.C230.21.013.

## Conflict of Interest

The authors declare that the research was conducted in the absence of any commercial or financial relationships that could be construed as a potential conflict of interest.

## Publisher's Note

All claims expressed in this article are solely those of the authors and do not necessarily represent those of their affiliated organizations, or those of the publisher, the editors and the reviewers. Any product that may be evaluated in this article, or claim that may be made by its manufacturer, is not guaranteed or endorsed by the publisher.
